# Genetic Variants Were Associated With the Prognosis of Head and Neck Squamous Carcinoma

**DOI:** 10.3389/fonc.2020.00372

**Published:** 2020-03-20

**Authors:** Yingzheng He, Pei Ji, Yuancheng Li, Ruixia Wang, Hongxia Ma, Hua Yuan

**Affiliations:** ^1^Jiangsu Key Laboratory of Oral Diseases, Nanjing Medical University, Nanjing, China; ^2^Department of Oral and Maxillofacial Surgery, Affiliated Hospital of Stomatology, Nanjing Medical University, Nanjing, China; ^3^Jiangsu Key Laboratory of Cancer Biomarkers, Prevention and Treatment, Cancer Center, Department of Epidemiology and Biostatistics, School of Public Health, Nanjing Medical University, Nanjing, China

**Keywords:** head and neck squamous cell cancer, single nucleotide polymorphism, cancer survival, cox regression, genetic variant

## Abstract

**Background:** As the sixth most common cancer of worldwide, head and neck cancers (HNC) are springing from oral cavity, pharynx and larynx and there is no strong biomarker for prognosis. Rates of 5 years survival with HNC remain relatively low in decades with improvement of treatments. Evidence that single nucleotide polymorphisms (SNPs) play a part in cancer prognosis is growing.

**Methods:** We conducted an exome-wide association study among 261 patients with head and neck squamous cell carcinoma (HNSCC) and then validated in The Cancer Genome Atlas (TCGA) database for survival by using the Cox proportional hazards regression models and Kaplan–Meier analyses.

**Results:** After combining the result of the two stages, 4 SNPs were significantly associated with HNSCC survival (rs16879870 at 6q14.3: adjusted HR = 2.02, 95%CI = 1.50–2.73, *P* = 3.88 × 10^−6^; rs2641256 at 17p13.2: adjusted HR = 0.67, 95%CI = 0.56–0.80, *P* = 7.51 × 10^−6^; rs2761591 at 11p13: adjusted HR = 2.07, 95%CI = 1.50–2.87, *P* = 1.16 × 10^−5^; and rs854936 at 22q11.21: adjusted HR = 1.92, 95%CI = 1.43–2.57, *P* = 1.27 × 10^−5^). Besides, we constructed a receiver operating characteristic (ROC) model to estimate predictive effect of the novel SNPs combined with clinical stage in HNSCC prognosis (AUC = 0.715). We also found the genotype of rs16879870 and rs854936 was significantly associated with the expression of gene *GJB7* (*P* = 0.013) and *RTN4R* (*P* = 0.047) in cancer tissues of TCGA, respectively.

**Conclusion:** Our findings suggested that the SNPs (rs16879870, rs2641256, rs2761591, rs854936) might play a crucial role in prognosis of HNSCC.

## Background

Head and neck cancer (HNC) is a heterogeneous disease that includes cancers involving the oral cavity (40%), pharynx (25%), and larynx (15%), which accounts for more than 550,000 new cases and 380,000 deaths each year around the world (1). Over 90% of head and neck cancers are squamous cell carcinomas (HNSCC). The estimated 5-year survival rate for HNSCC is appropriately 50% (2). Although advances in the understanding and treatment of HNSCC, there are still a large part of patients suffering from poor survival, with high rates of recurrence or metastasis (3, 4).

Risk factors for overall survival of HNSCC have been found recent years, including clinical staging, alcohol consumption, tobacco use, and HPV infection (5–7). HNC was historically treated with surgery and/or radiation (8). After treatment, smokers are at higher risk for treatment failure, disease recurrence, and development of second primaries (9–11). Tumor characteristics (e.g., location and size) and the patients' demographics (e.g., age) can also influence survival outcomes (12). Accumulating evidence suggests that genetic factors may also effect the survival of HNSCC (13). Single nucleotide polymorphisms (SNPs), the most common genetic variants, were known to all that they could contribute to drug response and to susceptibility to disease, including cancer. Several risk loci that associated with head and neck cancer were identified by many researches while prognostic factors were less well-studied. Wilkins et al. revealed that microRNA-related genetic variants were associated with overall HNSCC survival (14). SNPs in the genes of XPD and FGFR4 were also suggested to be possible predictors for overall survival after radiotherapy (15). Recently, the advances of high-throughput genotyping have provided a powerful tool to discover novel loci and genes for cancer prognosis.

In this study, we investigated the association between exome-wide genetic variants and outcomes in HNSCC by using Illumina Human Exome Bead Chip (Illumina, San Diego, CA). The Exome Chip contains more than 240,000 single nucleotide polymorphisms across exonic regions and adjacent areas (16). We first evaluated the association of SNPs from the exome chip with survival of HNSCC patients and then validated our findings based on The Cancer Genome Atlas (TCGA) database. The results will help to distinguish patients going through poor survival from all patients with HNSCC.

## Materials and Methods

### Study Populations

In the discovery stage, HNSCC patients were enrolled from Jiangsu Stomatological Hospital and the First Affiliated Hospital of Nanjing Medical University since January 2009 to May 2013. Five hundred and seventy-six patients with HNSCC were confirmed after diagnoses by two local pathologists. Patients with previous tumor in other organs or a history of chemotherapy or radiotherapy were removed. Clinical information was obtained through medical record while demographic and exposure data (e.g., tobacco smoking and alcohol consumption) were collected using the standard questionnaire through the face-to-face interview. Individuals who smoked an average of one cigarette or more per day for at least 1 year were defined as ever-smoking; otherwise, patients were considered as never-smoking. We considered people who had more than one alcoholic drink per day as drinkers. Five milliliter venous blood was collected from participants and genomic DNA was extracted with phenol chloroform. We connected with all participants by personal or family telephone contacts every 3 months from the time of recruitment until death or last time of follow-up (last follow-up in Mar 2018). Treatment information (radiotherapy or chemotherapy), metastasis or recurrence, and survival status (alive or dead, time of death, and cause of death) were included in the follow-up data. Furthermore, we also referred to the latest medical records as a complement. Finally, a total of 261 patients with full clinical and survival data were collected in this study. The study was approved by the institutional review board of Nanjing Medical University.

### Quality Control

Genotypes of 247,870 variants were called by Illumina Genome Studio software. It's necessary to manually check cluster plots. As described previously (Supplementary Figure 1), a systematic QC workflow was used to filter unqualified samples and genetic variants from the raw genotyping data. We removed individuals with low call rates (95%), extreme heterozygosity rates and familial relationships. We excluded variants if they (1) were mitochondrial variants, (2) were located out of autosomal chromosomes, (3) had duplicate variants, (4) were monomorphic in our study subjects, (5) had a call rate of <95%, (6) had a *P* < 1 × 10^−4^ for the Hardy Weinberg equilibrium (HWE) or (7) had a MAF <0.01. After quality control, 31,075 variants in 261 patients remained for further analysis.

### Replication Analysis

In order to reduce false-positive associations, promising variants were then selected for further replication based on The Cancer Genome Atlas (TCGA) database. We downloaded the clinical and survival data for HNSCC patients in TCGA. The original genotype data (level 1, Affymetrix 6.0 array) of 525 HNSCC patients were obtained in December 2014. The effect of SNPs and gene expression on survival was evaluated by multivariate Cox regression models with adjustment for age, sex, smoking, drinking, and clinical stage. In both our data and TCGA dataset, variants validated by the consistent associations with a *p* < 0.05 were considered to be significantly associated SNPs.

### eQTL and Function Analyses

We performed eQTL and function analyses based on TCGA database. The level 3 gene-level RNA-seq expression data of 497 HNSCC samples including 41 pairs were obtained from TCGA Firehose at the MIT Broad Institute (https://confluence.broadinstitute.org/display/GDAC/Home, 2014-12-18 release) and the level 1 genotyping data were also applied. Then, we performed expression quantitative trait loci (eQTL) analysis by using linear regression model with adjustment for age, gender, smoking, and drinking status in 497 HNSCC tissues. We also conducted differential expressed analysis between the tumors and adjacent normal tissues. Paired Wilcoxon rank sum test was performed to evaluate the differential expression of target genes in 41 tumor/adjacent pairs. The expression values of candidate genes were log_2_-transformed. Further survival analysis of target genes was constructed based on mRNA expression data and compatible clinical data of 497 HNSCC patients by using log-rank tests.

### Statistical Analysis

Days from the initial diagnosis of HNSCC to the death or the last follow-up was defined as the survival time. The crude or adjusted hazard ratio (HR) and their 95% confidence intervals (95% CI) for the associations of demographic and clinical variables with survival time were calculated by univariate Cox regression analysis using the survival package of R software. The associations between SNPs and HNSCC survival (in an additive genetic model) were analyzed by multivariate Cox regression models as previously described. A fixed-effects model of meta-analysis was used to combine the effects of discovery and replication stages when no heterogeneity was found between the two studies (Q > 0.05 and *I*^2^ < 25.0%); otherwise, a random-effects model was applied. The number of risk genotypes (NRG) was used as a genetic score to estimate the combined effect of all independent and significant SNPs. The difference of survival time between different subgroups sorted by genotypes or NRG were assessed by Kaplan-Meier curves and log-rank tests. The heterogeneity test of associations between subgroups in stratified analyses was performed by using the Q-test. Receiver operating characteristic (ROC) curve was constructed from the logistic regression model, and the area under the curve (AUC) was used to assess the classification performance of the model. Further gene function annotation for selected SNPs were based on biological information databases, such as TCGA, UCSC, and HaploReg.

## Result

### Patients' Characteristics and Clinical Features

The median survival time was 55.02 months, and 71 patients in our cohort (27.2%) died of head and neck cancer. 261 patients with primary HNSCC were contained in the discovery stage and clinical variables were shown in Supplementary Table 1, and 487 patients were included in the replication stage with clinical variables exhibited in Supplementary Table 1 as well. There were more men than women in the discovery stage as well as in the replication stage (59 vs. 41% and 73.7 vs. 26.3% in the discovery and replication stage, respectively). None of the clinical variables (age, gender, smoking status, drinking status, and clinical stage) was significantly associated with patients' survival time (*P* > 0.05), both in the discovery and replication stage. The small sample size of our study might be the reason for statistically non-significant results of clinical stage.

### Multivariate Analyses on Associations Between SNPs and HNSCC

As shown in Supplementary Figure 1, after QC, we performed Cox proportional hazards regression models to assess associations of 31,075 qualified genetic variants with HNSCC survival, with adjustment for age, gender, smoking status, drinking status, and clinical stage. Total 367 variants were found with *P* < 0.01. Then 302 variants were selected for validation in the TCGA cohort for survival analysis because the other 65 variants were not available in TCGA dataset. Finally, 20 SNPs were individually significantly correlated with HNSCC patients' survival with both *P* < 0.05 and consistent associations. Moreover, according to no evidence of heterogeneity across two data, we applied fixed-effects model to combine the result of the two stages. As a consequence, 4 SNPs were still considered noteworthy (*P*_adjusted_ < 0.05), including rs16879870 at 6q14.3 (HR_adjusted_ = 2.02, 95%CI = 1.50–2.73, *P*_adjusted_ = 3.88 × 10^−6^), rs2641256 at 17p13.2 (HR_adjusted_ = 0.67, 95%CI = 0.56–0.80, *P*_adjusted_ = 7.51 × 10^−6^), rs2761591 at 11p13 (HR_adjusted_ = 2.07, 95%CI = 1.50–2.87, *P*_adjusted_ = 1.16 × 10^−5^), and rs854936 at 22q11.21 (HR_adjusted_ = 1.92, 95%CI = 1.43–2.57, *P*_adjusted_ = 1.27 × 10^−5^) (Table 1). The primary information and effect allele frequency (EAF) of the four selected SNPs in each stage were presented in Table 1 as well.

**Table 1:** Primary information and Meta analyses of selected SNPs.

**Chrpos**	**SNP**	**Allele**	**NJMU**			**TCGA**			**Meta**		**Func**
			**EAF**	**HR (95% CI)^**a**^**	***P^***a***^***	**EAF**	**HR (95% CI)^**a**^**	***P^***a***^***	**HR (95% CI)^**b**^**	***P*_meta^**b**^**	
6:87531746	rs16879870	A/C	0.088	2.12 (1.25–3.59)	5.09E−03	0.056	1.98 (1.38–2.85)	2.36E−04	2.02 (1.50–2.73)	3.88E−06	Intergenic
17:5126674	rs2641256	G/A	0.289	0.56 (0.37–0.84)	5.49E−03	0.670	0.69 (0.57–0.84)	2.73E−04	0.67 (0.56–0.80)	7.51E−06	Exonic
11:31329373	rs2761591	A/G	0.027	2.91 (1.34–6.30)	6.72E−03	0.040	1.93 (1.34–2.76)	3.48E−04	2.07 (1.50–2.87)	1.16E−05	Exonic
22:20260814	rs854936	C/A	0.050	2.35 (1.25–4.40)	7.86E−03	0.059	1.81 (1.30–2.53)	4.08E−04	1.92 (1.43–2.57)	1.27E−05	Intergenic

SNP, single nucleotide polymorphism; EAF, effect allele frequency; HR, hazards ratio.

a *Derived from Cox proportional hazards regression models with an adjustment for age, gender, smoke, drink status, and clinical stage*.

b *Derived from Fixed-effects model of Meta analyses to combine the effects of NJMU and TCGA cohort*.

**Figure 1:**
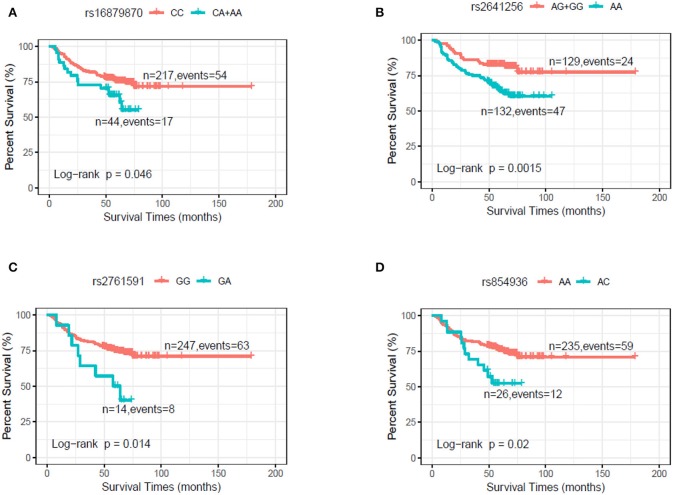
Kaplan–Meier plot by genotypes of selected SNPs in dominant model in the discovery stage. **(A)** rs16879870 (CC vs. CA+AA). **(B)** rs2641256 (AG+GG vs. AA). **(C)** rs2761591 (GG vs. GA). **(D)** rs854936 (AA vs. AC).

In the discovery stage, univariate and multivariable Cox regression analysis were further performed to evaluate the effects on risk of death for each of selected SNPs (Table 2), with adjusting of age, gender, smoking, drinking, and clinical stage. We found that the genotypes CA+AA of rs16879870 showed a significant association with a decreased survival time [HR = 2.12 (1.21–3.73), *P* = 0.008], compared to the genotype CC. The genotype AA of rs2641256 was associated with an increased death risk of HNSCC [AA vs. AG+GG: HR = 2.10 (1.28–3.46), *P* = 0.004]. Compared with the reference genotype, the patients with genotype GA of rs2761591 and AC of rs854936 had risks of overall death, respectively [rs2761591 GA vs. GG: HR = 2.91 (1.34–6.30), *P* = 0.007; rs854936 AC vs. AA: HR = 2.35 (1.25–4.40), *P* = 0.008]. The classification performance of risk genotypes of selected SNPs was illustrated by Kaplan-Meier curves (Figure 1).

**Table 2:** Associations between selected SNPs and survival time of HNSCC patients in discovery stage.

**Genotype**	**Frequency**		**Univariate analysis**		**Multivariate analysis**^****a****^	
	**All**	**Death (%)**	**HR (95% CI)**	***P***	**HR (95% CI)**	***P***
**rs16879870**						
CC	217	54 (24.88)	1.00		1.00	
CA	42	16 (38.10)	1.70 (0.97–2.97)	0.063	2.04 (1.15–3.62)	0.015
AA	2	1 (50.00)	1.58 (0.59–4.25)	0.366	2.43 (0.87–6.76)	0.089
CC	217	54 (24.88)	1.00		1.00	
CA+AA	44	17 (38.64)	1.73 (1.01–2.99)	0.049	2.12 (1.21–3.73)	0.008
**rs2641256**						
AA	132	47 (35.61)	1.00		1.00	
AG	107	20 (18.68)	0.46 (0.27–0.78)	0.004	0.49 (0.29–0.83)	0.008
GG	22	4 (18.18)	0.66 (0.40–1.11)	0.115	0.65 (0.39–1.09)	0.099
AG+GG	129	24 (18.60)	1.00		1.00	
AA	132	47 (35.61)	2.18 (1.33–3.56)	0.002	2.10 (1.28–3.46)	0.004
**rs2761591**						
GG	247	63 (25.51)	1.00		1.00	
GA	14	8 (57.14)	2.44 (1.17–5.11)	0.018	2.91 (1.34–6.30)	0.007
**rs854936**						
AA	235	59 (25.11)	1.00		1.00	
AC	26	12 (46.15)	2.06 (1.10–3.83)	0.023	2.35 (1.25–4.40)	0.008

*HR, hazards ratio; 95%CI, 95% confidence interval; HNSCC, head and neck squamous cell carcinoma*.

a *Derived from Cox proportional hazards regression models with an adjustment for age, gender, smoke, drink status, and clinical stage*.

To provide a better estimation of the hazards of survival, the risk genotypes (i.e., rs16879870 CA+AA, rs2641256 AA, rs2761591 GA, and rs854936 AC) were combined into one variable as the number of risk genotypes (NRG), which divided patients with HNSCC in the discovery stage into four groups: zero, one, two, and three risk genotypes. As shown in Table 3, we found the risk of death was improved with an increase of the number in NRG by using the multivariate analysis (trend test: *P* < 0.00001). Patients with 1-3 NRG raised the hazard of death of HNSCC by nearly fourfold [HR = 3.47 (1.90–6.37), *P* < 0.0001] and patients with 2–3 NRG increased the risk of death by nearly 2-fold [HR = 2.36 (1.63–3.41), *P* < 0.00001], compared with those who had no NRG. Kaplan-Meier survival curves were present to illustrate the associations between NRG and survival time (Supplementary Figure 2).

**Table 3:** Associations between combined NRG and survival time of HNSCC patients in discovery stage.

**NRG^**b**^**	**No**.		**Univariate analysis**		**Multivariate analysis**^****a****^	
	**Patients**	**Death (%)**	**HR (95% CI)**	***P***	**HR (95% CI)**	***P***
0	103	13 (12.62)	1.00		1.00	
1	106	36 (33.96)	3.11 (1.65–5.86)	<0.001	2.88 (1.52–5.46)	0.001
2	46	18 (39.13)	1.96 (1.37–2.82)	<0.001	2.29 (1.56–3.36)	<0.0001
3	6	4 (66.67)	1.89 (1.29–2.75)	<0.001	1.98 (1.28–3.06)	0.002
Trend				<0.0001		<0.00001
0	103	13 (12.62)	1.00		1.00	
1	106	36 (33.96)	3.11 (1.65–5.86)	<0.001	2.88 (1.52–5.46)	0.001
2–3	52	22 (42.31)	2.05 (1.45–2.90)	<0.0001	2.36 (1.63–3.41)	<0.00001
Trend				<0.0001		<0.00001
0	103	13 (12.62)	1.00		1.00	
1–3	158	58 (36.71)	3.44 (1.88–6.28)	<0.0001	3.47 (1.90–6.37)	<0.0001

*HR, hazards ratio; 95%CI, 95% confidence interval; NRG, number of risk genotypes; HNSCC, head and neck squamous cell carcinoma*.

a *Derived from Cox proportional hazards regression models with an adjustment for age, gender, smoke, drink status, and clinical stage*.

b *Risk genotypes were rs16879870 CA+AA, rs2641256 AA, rs2761591 GA, and rs854936 AC*.

### ROC Curve

**Figure 2:**
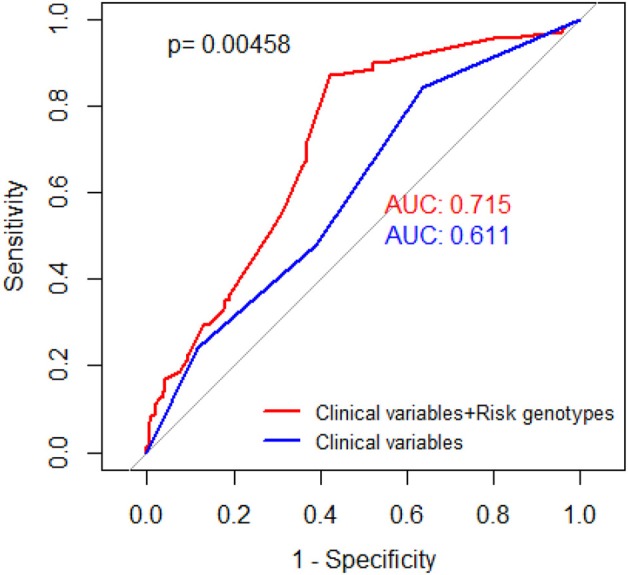
Receiver operating characteristic (ROC) curves. Red curve for prediction of HNSCC-specific survival rate based on selected SNPs and clinical stage, with adjustment of age, gender, smoking status, and drinking status (AUC = 0.715). Blue curve for prediction of HNSCC-specific survival rate based on clinical stage, with adjustment of age, gender, smoking status, drinking status (AUC = 0.611). *P* value was derived from DeLong's test for two correlated ROC curves.

ROC model was built to assess the ability of NRG in prediction of HNSCC survival by using the area under the curve (AUC). We constructed two models to compare their ability, one for clinical variables and the other for both clinical variables and all risk genotypes. As shown in Figure 2, with the addition of NRG, the prediction model of survival increased the AUC from 0.611 to 0.715.

### Stratified Analysis of Selected SNPs in the Discovery Stage

Furthermore, stratification analyses were conducted to examine whether the effects of risk genotypes on death with HNSCC was modified by sex, age, smoking, drinking status, and clinical stage. However, the heterogeneity test of subgroups by each of clinical feathers, except drinking status for rs2761591, were all significant (*P* < 0.05) (Supplementary Table 2).

### The eQTL and Function Analyses

**Figure 3:**
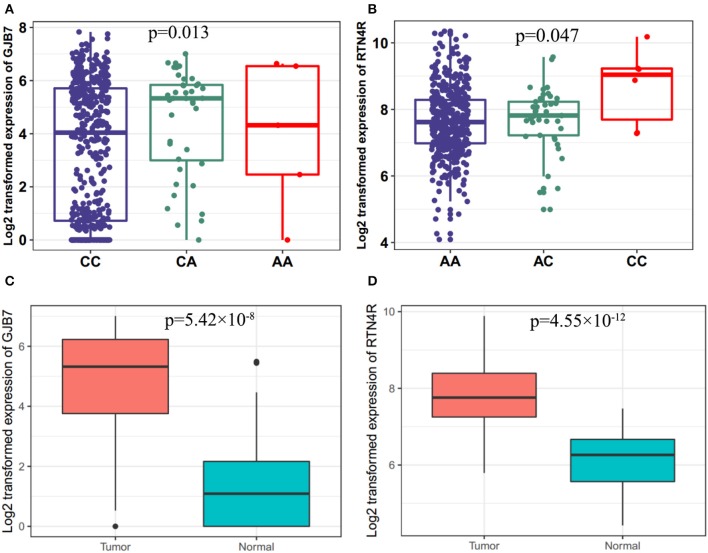
Associations between the risk genotypes and their corresponding mRNA expression levels. **(A)** The eQTL for gene *GJB7* and rs16879870 (*P* = 0.013) **(B)** eQTL for gene *RTN4R* and rs854936 (*P* = 0.047) in cancer tissues of TCGA in the additive model. eQTL, expression quantitative trait loci analysis. Differential expression analysis for **(C)** *GJB7* (*P* = 5.42 × 10–8) and **(D)** *RTN4R* (*P* = 4.55 × 10–12) in the TCGA dataset.

To further explore potential functions of these SNPs, we performed the eQTL analysis for selected SNPs and mRNA expression of their corresponding genes in cancer tissues by using TCGA dataset. As shown in Figure 3, the allele A of rs16879870 was statistically significantly associated with an increased mRNA expression level of gene *GJB7* (a trend test in an additive model: *P* = 0.013) and the allele C of rs854936 was significantly associated with an increased mRNA level of gene *RTN4R* (a trend test in an additive model: *P* = 0.047) (Figures 3A,B). We also assessed the influence of the two corresponding target genes on HNSCC survival by examining mRNA expression from 41 paired HNSCC tumor and normal tissues and evaluating the association between mRNA expression levels and survival time of HNSCC patients in TCGA dataset. The gene expression of *GJB7* and *RTN4R* in cancer tissues were obviously higher than normal tissues (*P* = 5.42 × 10^−8^ and 4.55 × 10^−12^ for *GJB7* and *RTN4R*, respectively) (Figures 3C,D). Using the mean expression values as the cutoff, the higher expression of *GJB7* and *RTN4R* had a significant worse prognosis in patients with HNSCC, respectively. (*P* = 0.029 and 0.018 for *GJB7* and *RTN4R*, respectively) (Supplementary Figure 3).

## Discussion

In the study, we investigated several potential genetic variants for predicting survival outcomes in discovery cohort and validated in TCGA cohort, and found that 4 SNPs (rs16879870, rs2641256, rs2761591, and rs854936) might be independent prognostic factors for HNSCC survival. In addition, among the four significant genetic variants, only two SNPs had corresponding target genes by using eQTL analysis and further functional analyses of SNP-target genes demonstrated that rs16879870-target *GJB7* and rs854936-target *RTN4R* might be promising risk factors for the prognosis of HNSCC.

By annotating in UCSC, we find that two expression quantitative trait loci (rs16879870 and rs854936) are intergenic SNP and other two genetic variants (rs2641256 and rs2761591) are located in exome region. Rs16879870 located between gene SNHG5 and HTR1E is ~460 kb away from target gene *GJB7* in chromosome 6. The risk allele A of rs16879870 increased the expression of *GJB7* and high expression of *GJB7* promotes bad outcomes of patients with HNC. GJB7 (gap junction protein beta 7) is a member of connexins which help intercellular signals to quickly travel between two adjacent cells (17). Studies have proved that GJB7 was a specific gap junction protein associated with cell communication and disrupted by RNAi could increase chemotherapy sensitivity in leukemia (18). These findings suggested GJB7 might play an important role in the prognosis of HNSCC and future studies could be aimed at determining if GJB7 is linked to HNSCC survival.

Rs854936 located within 22q11.21 is about 5 kb away from target gene *RTN4R*. The risk allele C of rs854936 significantly raised the expression of *RTN4R* and high expression of *RTN4R* decreased the rate of HNC survival. RTN4R (reticulon 4 receptor), which is an essential role in axonal regeneration and structural plasticity in the central nervous system (19, 20), has been elucidated to be associated with the risk for sporadic amyotrophic lateral sclerosis (21, 22) and schizophrenia (23, 24). That findings indicated that RTN4R might be a new risk factor for predicting cancer prognosis. However, the mechanism of either *GJB7* or *RTN4R* underlying the effect on the survival was not identified both in our study and in the literature.

Within the exon region of gene SCIMP, rs2641256 encodes a synonymous mutation (A>G). SCIMP (SLP adaptor and CSK interacting membrane protein), mainly expressed in antigen-presenting cells, is an important regulator of host innate immune responses (25) and could promote pro-inflammatory cytokine production from macrophages (26). Rs2761591 G>A is situated in the exon region of gene DCDC1 (doublecortin domain containing 1), which is mainly expressed in adult testis (27) and considered to be related with cell mitosis (28). There has other research found that DCDC1 was significantly associated with esophageal carcinoma as a mutated gene (29). However, neither SCIMP nor DCDC1 exhibited significantly differential expression in TCGA dataset.

Several potential limitations of the present study need to be considerate. First of all, a relatively small sample size may limit the statistical power of our study, especially in stratification analysis. Secondly, the loss of follow-up rate is about 54% in our study. However, in order to exclude withdraw bias, we compared the clinical data of follow-up and loss of follow-up by using *t* test and Chi-square test. As shown in Supplementary Table 3, most of clinical variables had no significant difference between two groups. Finally, the biological mechanism of the four SNPs was not confirmed and expanded in the research.

## Conclusion

Our study indicated that four SNPs (rs16879870, rs2641256, rs2761591, and rs854936) may have independent or joint effects on survival of HNSCC patients. However, our findings need to be validated in an independent, large-scale, and multiracial population and further function between SNPs and prognosis need to be evaluated.

## Data Availability Statement

Publicly available datasets were analyzed in this study, these can be found in the NCBI ClinVar database (https://www.ncbi.nlm.nih.gov/clinvar/) (accession: SCV001161482, SCV001161483, SCV001161484, and SCV001161485).

## Ethics Statement

The collection and use of the samples were reviewed and approved by the Institutional Ethics Committee of Nanjing Medical University. All subjects gave written informed consent in accordance with the Declaration of Helsinki.

## Author Contributions

HY and HM designed the study. PJ and HY collected samples and followed up patients. HY and RW finished functional SNPs selection and genotyping. YL and RW finished bioinformatics and statistic analysis. YH and PJ drafted the manuscript. All authors read and approved the final manuscript.

### Conflict of Interest

The authors declare that the research was conducted in the absence of any commercial or financial relationships that could be construed as a potential conflict of interest.

## Abbreviations

HNSCC: head and neck squamous cell carcinoma
SNPs: single nucleotide polymorphisms
HR: Hazard Ratio
CI: confident interval
NRG: number of risk genotypes.
