# Supplementary material for: Genetic Variants Were Associated With the Prognosis of Head and Neck Squamous Carcinoma
Source: Front Oncol. 2020 Mar 20;10:372. doi: 10.3389/fonc.2020.00372 (PMC7099049; doi:10.3389/fonc.2020.00372)
Supplement: Supplementary file 1 [file Table_1.DOC]

**Supplementary Table 1. Patients’ characteristics in discovery stage (NJMU) and replication stage (TCGA)**

| **Variables** | **Patients** | **Deaths** | **mean survival time** | ***P***^a^ | **HR (95% CI)**^a^ |
| --- | --- | --- | --- | --- | --- |
|  | **N=261 (%)** | **N=71** | **(month)** |  |  |
| **NJMU** |  |  |  |  |  |
| **Age** |  |  |  | 0.375 |  |
| ＜62 | 129 (49.4) | 31 | 54.72 |  | 1 |
| ≥62 | 132 (50.6) | 40 | 55.31 |  | 1.24 (0.77-1.98) |
| **Gender** |  |  |  | 0.697 |  |
| Female | 107 (41.0) | 27 | 53.35 |  | 1 |
| Male | 154 (59.0) | 44 | 56.18 |  | 1.10 (0.68-1.78) |
| **Smoking** |  |  |  | 0.114 |  |
| Never | 154 (59.0) | 36 | 56.54 |  | 1 |
| Ever | 107 (41.0) | 35 | 52.83 |  | 1.46 (0.91-2.32) |
| **Drinking** |  |  |  | 0.814 |  |
| Never | 159 (60.9) | 44 | 55.38 |  | 1 |
| Ever | 102 (39.1) | 27 | 54.46 |  | 0.94 (0.58-1.53) |
| **Clinical stage** |  |  |  | 0.167 |  |
| I/II | 153 (58.6) | 37 | 56.87 |  | 1 |
| III/IV | 108 (41.4) | 34 | 52.40 |  | 1.39 (0.87-2.21) |
| **TCGA** |  |  |  |  |  |
| **Age** |  |  |  | 0.063 |  |
| ＜61 | 242 (49.7) | 87 | 29.48 |  | 1 |
| ≥61 | 245 (50.3) | 119 | 31.64 |  | 1.30 (0.99-1.72) |
| **Gender** |  |  |  | 0.052 |  |
| Male | 359 (73.7) | 142 | 30.74 |  | 1 |
| Female | 128 (26.3) | 64 | 30.09 |  | 1.34 (1.00-1.81) |
| **Smoking** |  |  |  | 0.338 |  |
| Never | 117 (24.0) | 42 | 29.45 |  | 1 |
| Ever | 370 (76.0) | 164 | 30.92 |  | 1.18 (0.84-1.66) |
| **Drinking** |  |  |  | 0.785 |  |
| Never | 152 (31.2) | 69 | 32.38 |  | 1 |
| Ever | 335 (68.8) | 137 | 29.74 |  | 0.96 (0.72-1.29) |
| **Clinical stage** |  |  |  | 0.262 |  |
| I/II | 110 (22.6) | 44 | 33.38 |  | 1 |
| III/IV | 377 (77.4) | 162 | 29.75 |  | 1.21 (0.87-1.70) |

HR= hazards ratio; 95% CI = 95% confidence interval

^a^ Derived from Univariate Cox regression models

**Supplementary Table 2**. Stratification analysis of 4 identified SNP genotypes and HNSCC survival.

| Variables | rs16879870 (Patients/Deaths) | | | Adjusted HR | *P*^b^ for heterogeneity | | rs2641256 (Patients/Deaths) | | | | Adjusted HR | | *P*^b^ for heterogeneity | |
| --- | --- | --- | --- | --- | --- | --- | --- | --- | --- | --- | --- | --- | --- | --- |
|  | CC | CA | AA | (95% CI) *^a^* | |  | | AA | AG | GG | | (95% CI) *^a^* | |  |
| Age |  |  |  |  | | **3.37E-02** | |  |  |  | |  | | **5.10E-03** |
| <62 | 85/23 | 13/7 | 0/1 | 1.78 (0.85-3.74) | |  | | 41/23 | 48/7 | 9/1 | | 0.35 (0.17-0.73) | |  |
| ≥62 | 78/31 | 13/9 | 1/0 | 1.74 (0.83-3.62) | |  | | 44/24 | 39/13 | 9/3 | | 0.77 (0.46-1.31) | |  |
| Gender |  |  |  |  | | **5.60E-03** | |  |  |  | |  | | **1.58E-02** |
| Male | 93/36 | 16/8 | 0/1 | 1.24 (0.57-2.69) | |  | | 50/32 | 54/10 | 6/2 | | 0.46 (0.26-0.84) | |  |
| Female | 70/18 | 10/8 | 1/0 | 3.53 (1.68-7.41) | |  | | 35/15 | 33/10 | 12/2 | | 0.66 (0.36-1.18) | |  |
| Smoking |  |  |  |  | | **6.60E-03** | |  |  |  | |  | | **5.90E-03** |
| Non-smoker | 99/25 | 18/10 | 1/1 | 2.39 (1.24-4.59) | |  | | 50/19 | 54/16 | 14/1 | | 0.61 (0.36-1.03) | |  |
| Smoker | 64/29 | 8/6 | 0/0 | 1.62 (0.65-4.04) | |  | | 35/28 | 33/4 | 4/3 | | 0.47 (0.23-0.94) | |  |
| drinking |  |  |  |  | | **5.10E-03** | |  |  |  | |  | | **6.60E-03** |
| Non-drinker | 98/32 | 17/11 | 0/1 | 2.49 (1.32-4.72) | |  | | 48/27 | 53/14 | 14/3 | | 0.55 (0.34-0.90) | |  |
| Drinker | 65/22 | 9/5 | 1/0 | 1.51 (0.58-3.95) | |  | | 37/20 | 34/6 | 4/1 | | 0.58 (0.26-1.30) | |  |
| Clinical stage |  |  |  |  | | **8.70E-03** | |  |  |  | |  | | **4.50E-03** |
| I&II | 99/26 | 16/10 | 1/1 | 2.57 (1.31-5.02) | |  | | 49/25 | 58/9 | 9/3 | | 0.48 (0.26-0.87) | |  |
| III&IV | 64/28 | 10/6 | 0/0 | 1.36 (0.54-3.44) | |  | | 36/22 | 29/11 | 9/1 | | 0.62 (0.34-1.12) | |  |

| Variables | rs2761591 (Patients/Deaths) | | | Adjusted HR | *P*^b^ for heterogeneity | rs854936 (Patients/Deaths) | | | Adjusted HR | *P*^b^ for heterogeneity |
| --- | --- | --- | --- | --- | --- | --- | --- | --- | --- | --- |
|  | GG | GA | AA | (95% CI) *^a^* |  | GG | GA | AA | (95% CI) *^a^* |  |
| Age |  |  |  |  | **1.32E-02** |  |  |  |  | **5.30E-03** |
| <62 | 95/3 | 89/3 | - | 3.39 (0.98-11.74) |  | 89/9 | 87/5 | - | 1.95 (0.73-5.22) |  |
| ≥62 | 28/3 | 35/5 | - | 2.28 (0.85-6.10) |  | 26/5 | 33/7 | - | 2.97 (1.28-6.87) |  |
| Gender |  |  |  |  | **6.00E-03** |  |  |  |  | **6.30E-03** |
| Male | 106/39 | 39/5 | - | 5.25 (1.42-19.39) |  | 74/21 | 6/6 | - | 3.51 (1.35-9.12) |  |
| Female | 78/24 | 2/3 | - | 2.18 (0.83-5.70) |  | 102/38 | 8/6 | - | 1.81 (0.76-4.31) |  |
| Smoking |  |  |  |  | **2.60E-03** |  |  |  |  | **8.40E-03** |
| Non-smoker | 116/31 | 2/5 | - | 5.23 (2.25-17.24) |  | 107/29 | 11/7 | - | 2.42 (1.04-5.65) |  |
| Smoker | 68/32 | 4/3 | - | 1.35 (0.40-4.54) |  | 69/30 | 3/5 | - | 2.27 (0.87-5.93) |  |
| drinking |  |  |  |  | 2.19E-01 |  |  |  |  | **6.00E-03** |
| Non-drinker | 114/39 | 1/5 | - | 7.26 (2.62-20.12) |  | 107/36 | 8/8 | - | 2.52 (1.16-5.48) |  |
| Drinker | 70/24 | 5/3 | - | 1.19 (0.35-4.03) |  | 69/23 | 6/4 | - | 2.25 (0.76-6.72) |  |
| Clinical stage |  |  |  |  | **2.80E-03** |  |  |  |  | **6.20E-03** |
| I&II | 113/31 | 3/6 | - | 5.20 (2.09-12.93) |  | 106/31 | 10/6 | - | 2.28 (0.94-5.56) |  |
| III&IV | 71/32 | 3/2 | - | 0.97 (0.22-4.23) |  | 70/28 | 4/6 | - | 2.61 (1.05-6.49) |  |

HR= hazards ratio; 95%CI= 95% confidence interval.

^a^ Derived from Stratification analysis of genotypes and HNSCC survival

^b^ P for heterogeneity: P value for heterogeneity by cochrane’s Q test

**Supplementary Table 3. Demographic difference between follow-up and loss of follow-up.**

| **Variables** | **Follow-up** | **Loss of follow-up** | **t or X^2^ value^a^** | ***P*** |
| --- | --- | --- | --- | --- |
|  | **N=261 (%)** | **N=315 (%)** |  |  |
| **Age** | 61.97±10.99 | 60.42±10.70 | -1.71 | 0.089 |
| **Gender** |  |  | 3.00 | 0.083 |
| Female | 107(41.0) | 106 (33.7) |  |  |
| Male | 154(59.0) | 209 (66.3) |  |  |
| **Smoking** |  |  | 3.56 | 0.059 |
| Never | 154(59.0) | 160 (50.8) |  |  |
| Ever | 107(41.0) | 155 (49.2) |  |  |
| **Drinking** |  |  | 5.15 | 0.023 |
| Never | 159(60.9) | 160 (51.1) |  |  |
| Ever | 102(39.1) | 153 (48.9) |  |  |
| **Clinical stage** |  |  | 0.24 | 0.627 |
| I/II | 153(58.6) | 67 (55.4) |  |  |
| III/IV | 108(41.4) | 54 (44.7) |  |  |

^a^ t test was used to compare numerical variable (age) and Chi-square test was performed to compare categorical variables (gender, smoking, drinking and clinical stage).

**Excluding variants (n=216,795):**

Variants not in autosomal chromosomes (n=5,574);

Duplicated variants (n=831);

Monomorphic variants (n=193,759);

P<0.0001 for Hardy-Weinberg equilibrium test (n=363);

Call rata <95% (n=314);

Variant calling visually inspected without a clear genotyping cluster (n=1,196)

MAF<0.01 (n=14,758)

**Removing samples (n=0):**

Abnormal heterozygosity (n=0);

Genotype missing rate>5% (n=0);

Familial relationships (n=0)

**Selecting variants for replication:**

367 variants P-value<0.01 for survival analysis

**Discovery stage**

Exome arraygenotyping

for 247,870 variants in 261 cases

**Discovery stage**

31,075 variants passing quality control

in 261 cases

**Discovery stage**

31,075 variants in 261 cases

for survival analysis

**Replication stage**

302 variants in 487 cases from TCGA

for survival analysis

**Replication stage**

20 variants P-value<0.05

The same direction of survival analysis

**Meta analysis**

4 variants FDR adjusted P-value<0.05

**Supplementary Figure 1. Flow chart**

**
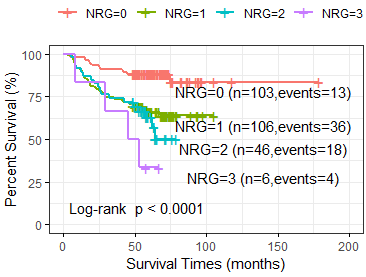
**


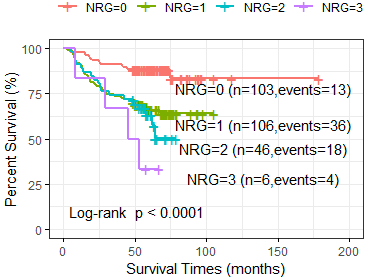

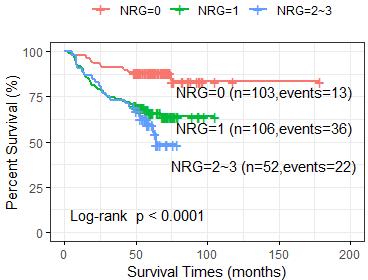

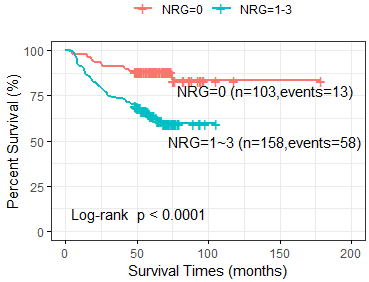


a b c

**Supplementary Figure 2. Kaplan–Meier analysis for patients with HNSCC by the combined number of risk genotypes (NRG). (a) By 0, 1, 2 and 3 risk genotypes (log-rank test: p<0.0001); (b) By 0, 1 and 2–3 risk genotypes (log-rank test: *p*<0.0001); and (c) by 0 and 1–3 risk genotypes (log-rank test: *p*<0.0001).**

a b


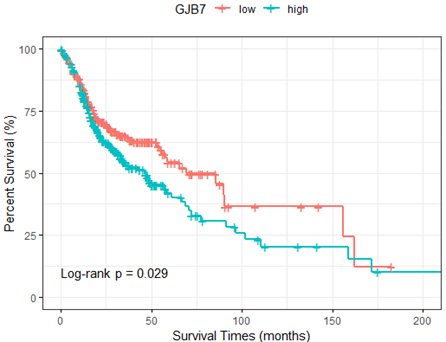

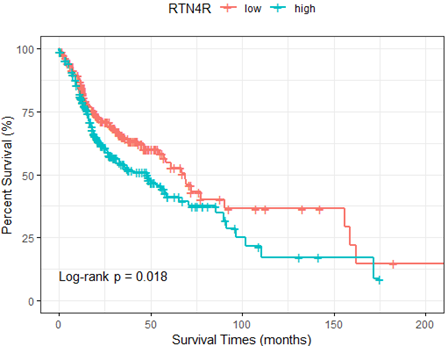


**Supplementary Figure 3. a) Kaplan–Meier plot for patients with HNSCC (TCGA) survival by *GJB7* (*P*=0.029) and b) *RTN4R* (*P*=0.018).**
